# Atomic-Scale Insights into the Deformation Mechanism of the Microstructures in Precipitation-Strengthening Alloys

**DOI:** 10.3390/ma16051841

**Published:** 2023-02-23

**Authors:** Chenshuang Wei, Sai Tang, Yi Kong, Xiong Shuai, Hong Mao, Yong Du

**Affiliations:** 1 State Key Lab for Powder Metallurgy, Central South University, Changsha 410083, China; 2 National Key Laboratory of Science and Technology on High-Strength Structural Materials, Central South University, Changsha 410083, China; 3 Hunan Institute of Science and Technology, College of Mechanical Engineering, Yueyang 414006, China

**Keywords:** deformation behavior, nano-precipitate, atomic scale simulation, precipitation-strengthening, phase field crystal method

## Abstract

Clarifying the deformation behaviors of microstructures could greatly help us understand the precipitation-strengthening mechanism in alloys. However, it is still a formidable challenge to study the slow plastic deformation of alloys at the atomic scale. In this work, the phase-field crystal method was used to investigate the interactions between precipitates, grain boundary, and dislocation during the deformation processes at different degrees of lattice misfits and strain rates. The results demonstrate that the pinning effect of precipitates becomes increasingly strong with the increase of lattice misfit at relatively slow deformation with a strain rate of 10^−4^. The cut regimen prevails under the interaction between coherent precipitates and dislocations. In the case of a large lattice misfit of 19.3%, the dislocations tend to move toward the incoherent phase interface and are absorbed. The deformation behavior of the precipitate-matrix phase interface was also investigated. Collaborative deformation is observed in coherent and semi-coherent interfaces, while incoherent precipitate deforms independently of the matrix grains. The faster deformations (strain rate is 10^−2^) with different lattice misfits all are characterized by the generation of a large number of dislocations and vacancies. The results contribute to important insights into the fundamental issue about how the microstructures of precipitation-strengthening alloys deform collaboratively or independently under different lattice misfits and deformation rates.

## 1. Introduction

It is well known that precipitation-strengthening is a ubiquitous strengthening mechanism for a wide range of materials [1,2,3]. The precipitation state-induced mechanism enhances the microhardness and strength of maraging steels [4,5]. In 2xxx and 7xxx series aluminum alloys, dispersed nano-precipitates determine the strength of alloys, making the two series aluminum alloys very important candidates of high-strength light-weight structural materials in aerospace industry [6,7,8]. Numerous dispersed nano-precipitates play the role of pinning grain boundaries (GBs) and dislocations during deformation [9,10,11]. They are especially important under the circumstances of deformation during alloy processing and service. In the last decades, the underlying mechanisms of the interactions between precipitate, dislocation, and GB has always arisen great interest from scientists [12,13,14,15]. Analytical models have been proposed to explain this issue macroscopically [11,16]. Though advanced electron microscope characterizations have greatly promoted our understanding on the mechanisms of microstructure evolution of deformation [17,18], we still know very limited about the atomic scale mechanisms during the deformation processes of the microstructures, including precipitates, GBs, dislocations, and matrix phase grains in alloys [19].

Essentially, the dispersed nano-precipitates strengthen alloys by pinning GBs and dislocations [8,20,21]. Previous works have verified that the movement of GBs could be hindered by the second-phase particles [22,23]. Furthermore, Tan et al. [24] found that the pinning force of precipitates relative to GBs is affected by the size of precipitates, the migration distance of GBs, and the misorientation angle of GBs. Nano-precipitates could also interact strongly with dislocations. Scientists regarded that dislocations overcome deformable precipitates via a cutting way, while impenetrable precipitates via Orowan looping [25,26]. However, this often leads to strain localization around the precipitates, which is the origin of the strength-ductility conflict in precipitation-strengthening alloys. The previous studies have greatly enriched our understanding of the interactions between precipitate and dislocation/GB; nevertheless, the atomic-scale mechanisms of such interactions are still unclear.

The precipitate-matrix interface plays a significant role in coordinating deformation of the precipitates and matrix. The fundamental problems on clarifying how the interface coordinates deformations have attracted lots of interest from scientists. The structure of the precipitate-matrix interface is determined by the lattice misfits between the precipitate and matrix phases, which strongly affects the deformation behavior of materials. Precipitation-strengthening alloys, such as aluminum alloys, are often characterized by a rich variety of precipitates with lattice misfits ranging from less than 1% to even 20% [2,27]. The lattice misfit *f* is calculated by $f=\left(a_{0}-a_{1}\right)∕a_{0}$, where $a_{0}$ and $a_{1}$ are the lattice constants of the matrix phase and precipitate, respectively. In recent years, it has been widely found that coherent or semi-coherent nanoprecipitates could profoundly increase the strength of a wide range of advanced alloys [5,28,29,30], including aluminum alloys, high entropy alloys, high-temperature alloys, and advanced steels. In contrast, incoherent precipitates are often regarded to be disadvantageous to the strength of alloys. Such a difference is essentially attributed to the deformation behavior of the precipitate-matrix phase interface with different coherencies. Clarifying the different deformation mechanisms of the precipitate-matrix interface is also the key to understanding the strengthening effect of precipitates.

However, it is still a formidable challenge to study the slow deformation process in real time, e.g., the slow stretching of nano-precipitates and grains of alloys, with the resolution up to the atomic scale in both experiments and simulations [31,32]. Phase-field simulations [33] are unable to fundamentally elucidate the mechanisms of microstructure evolution at the atomic scale. Molecular dynamics (MD) [34,35] simulations and first-principles calculations [31,36] have been used to study the dislocation-precipitate interactions, but none of these methods can reproduce the microstructure evolution during a slow plastic deformation process. Therefore, MD simulation could only simulate a very fast deformation process with a strain rate of up to 10^8^/s. In recent years, the phase-field crystal (PFC) model has naturally coupled all the physical properties generated by periodic structures and simulated the diffusive evolution of microstructures on the atomic spatial scale [37,38] during slow plastic deformation. The deformation of pure material systems has been successfully reproduced by PFC simulations [39,40].

In this work, the deformation behavior of precipitates in precipitation-strengthening alloys were investigated by using the PFC model [41,42]. We focused on the evolution of atomic configuration of microstructures, dislocation density, and free energy during the deformation process. Firstly, the deformation mechanisms of microstructure with different lattice misfits were analyzed under different strain rates ranging from 10^−4^ to 10^−2^. Then the deformation mechanisms of precipitate-matrix phase interface with different lattice misfits were also characterized. The results provide new insights at the atomic scale into the deformation mechanisms of alloys with precipitate-matrix structures.

## 2. Model and Simulation Details

### 2.1. Phase-Field Crystal Model

The PFC methods simulate the physical phenomena of atomic length scale and diffusion time scale by introducing periodic atomic density states to minimize the free energy. The PFC methods also capture the salient physics of diffusional phase transitions involving atomic scale elastic and plastic interactions. The free energy function of PFC model comes from the classical density functional theory [43]. The total free energy is written as,

(1) $$\frac{\Delta F}{k_{B}T\rho^{0}}=\int drf=\int dr\left\{\Delta F_{id}+\Delta F_{ex}\right\}$$

where $\Delta F_{id}$ and $\Delta F_{ex}$ are the dimensionless ideal mixture energy and excess energy, respectively, $\rho^{0}$ is the atom density of the reference state, $k_{B}$ is the Boltzmann constant, and T is the temperature [44]. The ideal free energy is given by,

(2) $$\Delta F_{id}=\int \left\{\left[1+n\left(\vec{r}\right)\right]ln\left[1+n\left(\vec{r}\right)\right]-n\left(\vec{r}\right)\right\}d\vec{r}\approx \int \left[\frac{n{\left(\vec{r}\right)}^{2}}{2}-\eta \frac{n{\left(\vec{r}\right)}^{3}}{6}+\chi \frac{n{\left(\vec{r}\right)}^{4}}{12}\right]d\vec{r}$$

where $n\left(\vec{r}\right)=\frac{\rho \left(\vec{r}\right)}{\rho_{0}}-1$ is the dimensionless atom density of the reference state, η and χ are parameters that correspond to an expansion about a particular reference state, both of which are adjustable to fit the ideal energy [45]. The excess free energy is written as,

(3) $$\Delta F_{ex}=-\frac{1}{2}\int n\left(\vec{r}\right)\int c_{2}\left(\left|\vec{r}-{\vec{r}}^{\prime }\right|\right)n\;\left({\vec{r}}^{\prime }\right)d{\vec{r}}^{\prime }d\vec{r}$$

where $C_{2}\left|\vec{r}-{\vec{r}}^{\prime }\right|$ is the direct correlation function.

The simplified dimensionless free energy functional reads as [45],

(4) $$\frac{\Delta F}{kT\rho^{0}}=\int \left\{\begin{array}{c} \frac{n^{2}}{2}-\eta \frac{n^{3}}{6}+\chi \frac{n^{4}}{12}+\left(n+1\right)\;\Delta F_{mix}-\\ \frac{1}{2}n\int dr^{\prime }C_{eff}^{n}\;\left(\left|r-r^{\prime }\right|\right)\;n^{\prime }+\alpha {\left|\vec{\nabla }c\right|}^{2} \end{array}\right\}dr$$

where $C_{eff}^{n}$ is the effective correlation function, and *α* is a coefficient that sets the energy of the compositional interface [46]. The mixing entropy $\Delta F_{mix}$ is expressed as follows:

(5) $$\Delta F_{mix}=\omega \left\{c\ln\;\left(\frac{c}{c_{0}}\right)+\left(1-c\right)\ln\;\left(\frac{1-c}{1-c_{0}}\right)\right\}$$

where the parameter ω is also used to fit the ideal energy. More details have been described previously [47].

Greenwood et al. [45] introduced $C_{eff}^{n}$, which is weighted by the composition field. It is given by

(6) $$C_{eff}^{n}=X_{1}\left(c\right)\;C_{2}^{AA}+X_{2}\left(c\right)C_{2}^{BB}$$

where $X_{1}\left(c\right)=1-3c^{2\;}+2c^{3}$,

(7) $$X_{2}\left(c\right)=1-3{\left(1-c\right)}^{2}+2{\left(1\;c\right)}^{3}$$

To be specific, a reciprocal space peak of $C_{2}^{ii}$ corresponding to mode *j* has the general form

(8) $${\hat{C}}_{2j}^{ii}=e^{-\frac{\sigma^{2}k_{j}{}^{2}}{2\rho_{j}\beta_{j}}}e^{-\frac{{\left(k-k_{j}\right)}^{2}}{2\alpha_{j}{}^{2}}}$$

where *ii* = AA and BB, σ is the effective temperature, $\rho_{j}$ is the atomic density of the plane, $\beta_{j}$ is the planar symmetry, and $\alpha_{j}$ is the width of the Gaussian peak. Specifically, varying $\alpha_{j}$ changes the width of a liquid-solid interface, which directly affects the surface energy and can control the magnitude of the elastic coefficients. [48].

The dynamic equations of the density and concentration fields can be written as

(9) $$\frac{\partial n}{\partial t}=\vec{\nabla }\cdot \left\{M_{n}\vec{\nabla }\left(\frac{\delta F}{\delta n}\right)\right\}$$

(10) $$\frac{\partial c}{\partial t}=\vec{\;\nabla }\cdot \;\left\{M_{c}\vec{\nabla }\left(\frac{\delta F}{\delta c}\right)\right\}$$

where $M_{n}$ and $M_{c}$ are the dimensionless kinetic mobility parameters, and t is time. In this study, we set the mobility coefficients to the constant 1 [47,49]. As for the $M_{n}$ and $M_{c}$, it is possible to efficiently evolve these dynamics java script in reciprocal space using semi-implicit techniques. The values of the parameters are given in Table 1 in terms of previous PFC simulation studies [44,45,46]. The density phase diagram shows that when the reference density $\rho_{0}$ is 0.01, the system reaches the most stable state [43,45]. The reference composition is taken as $c_{0}$ = 0.5 for the entropy of mixing [44,49]. According to solid and liquid energy curves, the fitting result is the best when the polynomial fitting parameters are η=1.4, χ=1 [49]. Setting the entropy of mixing coefficient ω=0.005 not only ensures that no pure material will appear, but also does not affect diffusion [44,49]. The selection of parameters for correlation function $\sigma_{M_{j}}\;\mathrm{and}\;\alpha_{i}$ is based on the temperature dependence of elastic coefficients [44,45].

Stefanovic et al. [50] introduced the modified phase field crystal (MPFC) model for materials deformations that takes the elastic strain into account. This model is capable of capturing fast dynamics up to acoustic velocity of deformation processes. The simplest equation of motion is,

(11) $$\frac{\partial^{2}n}{\partial t^{2}}+\beta \frac{\partial n}{\partial t}=\tau^{2}\nabla^{2}\frac{\delta F\left[n;T\right]}{\delta n}$$

where the dimensionless effective vacancy diffusion coefficient is β = 0.01 and the effective sound speed is τ = 0.05. The values of β and τ are set in terms of ref [50].

### 2.2. Simulation Details

**Figure 1:**
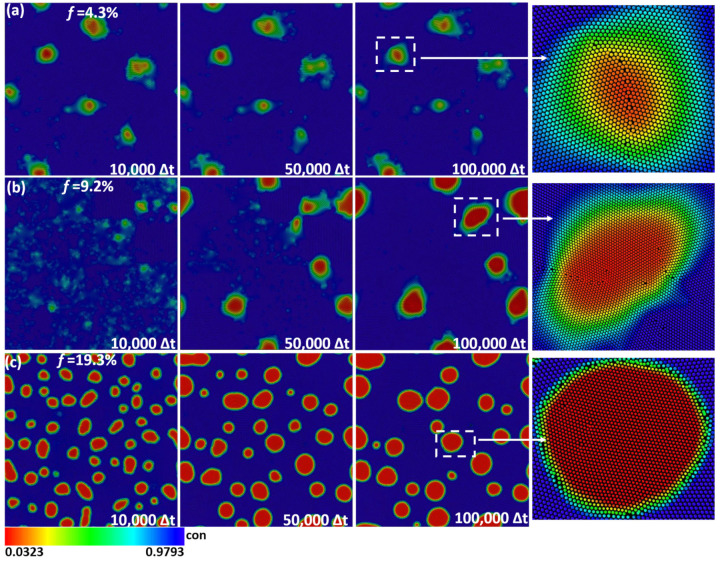
The evolution of precipitation process with different lattice misfits: (**a**) *f* = 4.3%; (**b**) *f* = 9.2%; (**c**) *f* = 19.3%. The deformation simulations are conducted after 100,000 Δt, as shown in the Videos S1–S3 in the Supplementary Materials. Note that the “con” on the right side of the color bar is the abbreviation for concentration.

The size of the simulation box is 2400 × 2400 grid spacing (equivalent to 300 × 300 atoms). Initially, four grains of the matrix phase are set in each of the X and Y directions; the grains are separated by a very thin liquid film. Two steps are conducted in our simulations. The first step is the precipitation simulation to produce the nano-precipitates from the initial polycrystalline matrix. After some iterations, the four initial separated grains grow together soon; then, the initial liquid films are replaced by GBs, and the precipitated phases nucleate and grow. Finally, the microstructures required for deformation are obtained as shown in Figure 1. For more details, please refer to our previous studies on the precipitation simulation of alloys [51,52]. The dimensionless lattice constants for matrix phase am = 1, and three lattice constants for precipitate phase *a*_p_ are 0.92 (lattice misfit *f* = 4.3%), 0.88 (*f* = 9.2%), and 0.8 (*f* = 19.3%), which, respectively, gives rise to coherent, semi-coherent, and incoherent interfaces. Figure 1 presents the precipitation process of nano-precipitates. The precipitates size and volume fraction are representative of real alloys [53,54]. The initial grid spacing $dx_{0}$ = $dy_{0}$ = 0.125 is to ensure that each atom is resolved by eight mesh spacing. The dynamical Equations (9)–(11) are solved semi-implicitly in Fourier space, and the time step Δt is set as 0.05 [55].

The second step is the deformation simulation. Dynamic Equation (11) is employed. The key is applying the load. The constant volume deformation model and the periodic boundary condition are adopted in our deformation simulations [56]. We conducted the deformation simulation of the microstructures of the precipitation process at 10^5^ Δt in Figure 1. Here, we set a tension force along the x direction with strain rate $\dot{\varepsilon_{x}}$, and simultaneously compression force along the y direction with strain rate $\dot{\varepsilon_{y}}$. At the kd time steps, the changed grid sizes in the x direction and the y direction are calculated as,

(12) $$dx=dx_{0}\cdot \left(1+\dot{\varepsilon }\cdot kd\cdot \Delta t\right)$$

(13) $$dy=dy_{0}∕\left(1+\dot{\varepsilon }\cdot kd\cdot \Delta t\right)$$

where the $\dot{\varepsilon }$ is the strain rate, and $dx_{0}$ and $dy_{0}$ are the initial grid sizes during the deformation. The area approximately satisfies $S=dx\cdot \;dy\approx dx_{0\;}\cdot dy_{0}$ during the deformation process. In this paper, strain rates $\dot{\varepsilon }$ are set as 10^−4^, 10^−3^ and 10^−2^. Therefore, the strain is $\varepsilon =\varepsilon_{x}-\varepsilon_{y}=\dot{\varepsilon }\;\cdot kd\cdot \Delta t.$

## 3. Results and Discussion

### 3.1. Deformation Mechanisms of Microstructure at Different Lattice Misfits

#### 3.1.1. Precipitate-GB Interactions

**Figure 2:**
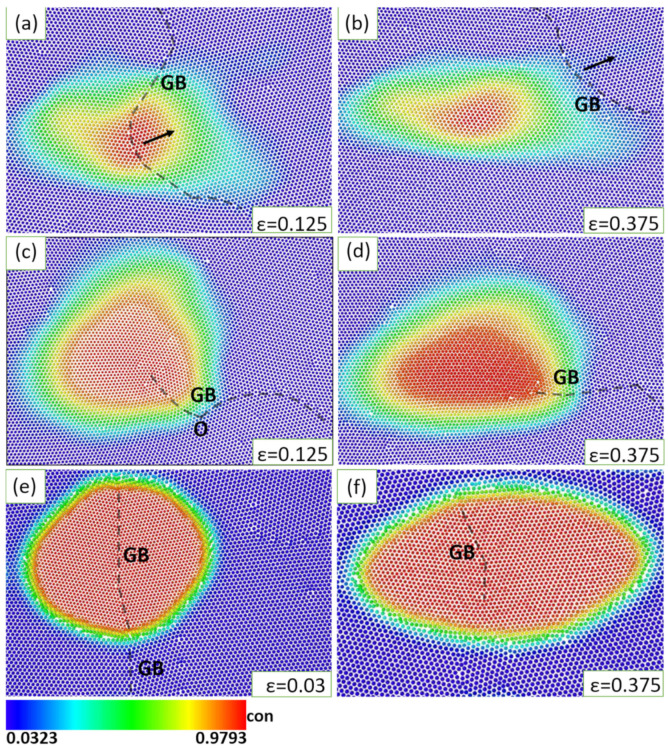
The interactions between GB and precipitate during slow deformation with strain rate $\dot{\varepsilon }$ = 10^−4^ with lattice misfit (**a**,**b**) *f* = 4.3%, (**c**,**d**) *f* = 9.2%, (**e**,**f**) *f* = 19.3%. Note that the time evolution of deformation process is characterized by the increase of applied deformation strain (ε). The black arrows in (**a**,**b**) are the direction of GB movement. For more details of the three deforming precipitates, please refer to Videos S4–S6 in the Supplementary Materials.

The GB-precipitate interactions were first investigated, and the strain rate was set at $\dot{\varepsilon }$ = 10^−4^. As we know, the second phases usually prefer to precipitate at GBs in alloys, which may profoundly affect the deformation behavior of GBs. In the case of a small lattice misfit of 4.3% (Figure 2a,b), the coherent precipitate-matrix interface structure exerts a weak force to pin the GB. As the deformation proceeds, the GB finally escapes from the precipitated phase and moves toward the matrix phase. In the case of the lattice misfit of 9.2% (Figure 2c,d), the obtained semi-coherent precipitate-matrix interface contains a bunch of misfit dislocations. The pinned GB penetrates through the precipitate-matrix interface, which releases the misfit strain of the semi-coherent interface. As shown in Figure 2c, the GB has been divided into two parts by the intersection point O, and bends sharply at this point due to deformation. Even though the pinned GB could migrate and slip during deformation, it is firmly pinned by the precipitate, and the two parts of the GB always connect. The situation is different from the case of a large lattice misfit of 19.2%. As shown in Figure 2e,f, the incoherent precipitate-matrix interface divides the GB into two parts, one segment in the precipitate and the other one in the matrix phase, but the two parts are totally separated by the precipitate/matrix interface and evolve independently. Moreover, the results above are compared with other deformation process of the GBs without precipitates simultaneously. Without the pinning of precipitates, under tensile deformation, these GBs move faster by rotation, straightening and merging, accompanied substantial changes of GB structure [57,58].

The results demonstrate that the pinning effect of precipitates can actually hinder the motion of deformed GBs, and becomes increasingly strong with the magnitude of lattice misfit. The interactions between GB and precipitate also changes with lattice misfit. Microscopically, the moving GB could cut through the coherent precipitates, but may be divided into several parts by incoherent precipitates.

#### 3.1.2. The Dislocation-GB/Precipitate Interactions

**Figure 3:**
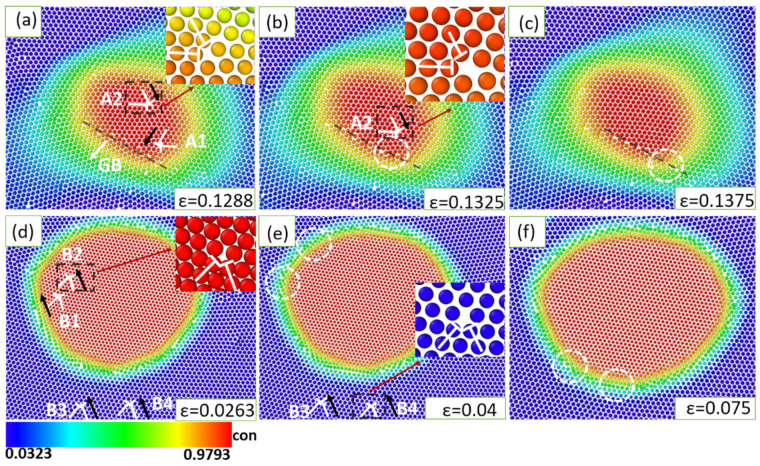
The dislocation dynamics around the deformed precipitate at different lattice misfits: (**a**–**c**) *f* = 4.3% (**d**–**f**) *f* = 19.3%. Note that the black arrows mark the moving directions of dislocation, the white circles are signs, the red arrows are the indicators.

In this section, the strain rate is fixed at $\dot{\varepsilon }$ = 10^−4^. Dislocation dynamics plays a crucial role in the deformation of precipitates and grains. As the deformation proceeds, a great number of dislocations are generated continuously from the deformed precipitates and grains, and subsequently are absorbed by GBs and precipitate-matrix phase interface. The coherent precipitate-matrix interface shows a very limited capacity to accommodate the generated dislocation during deformation. The moving dislocations have to penetrate through the precipitate/matrix interface quickly without a duration of stay, implying that the cut regimen prevails under the interaction between coherent precipitate and dislocations. This is in line with previous theories and studies that the coherent precipitate tends to be cut by moving dislocations [59,60]. They move toward the GBs and finally are absorbed as shown in Figure 3a–c. The dislocation pairs A1 are absorbed by the GB first, as shown in Figure 3a,b. As shown in Figure 3b,c, the dislocation pairs A2 are also absorbed by the GB. It can be observed that the two dislocations with opposite signs react and merge into a new dislocation, which then move towards the GB driven by the external forces during the evolution of the dislocation. In reverse, the GB can also emit dislocations continuously. Some of these dislocations move to other GBs and some are annihilated by the dislocations with the opposite burgers vector. This has also been reported in previous studies [61].

The dislocation dynamics is very different in the case of a large lattice misfit of 19.3%. The incoherent precipitate-matrix interface owns a much higher capacity to accommodate dislocations. Being different from in the case of 4.3% lattice misfit, the dislocations tend to move to the phase boundary and be absorbed. As shown in Figure 3d,f, the dislocation pairs B1, B2, B3, and B4 all are absorbed into the phase interface.

**Figure 4:**
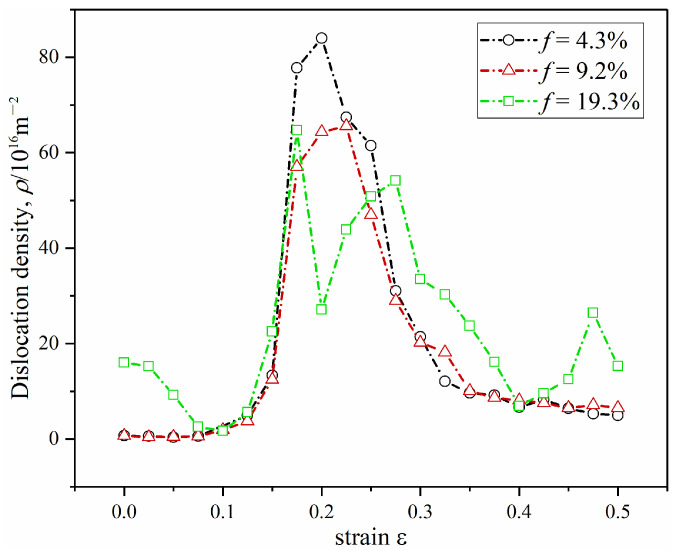
Dislocation density *ρ*-strain *ε* curves of the slow deformation process with $\dot{\varepsilon }$ = 10^−4^ at different lattice misfits of 4.3%, 9.2% and 19.3%.

Moreover, we quantified the change in dislocation density during the deformation process with the deformation strain of up to 50%. In the early stage of deformation (deformation strain < 20%), as the deformation of grains proceeds through elongation along the tensile direction or grain rotation, GBs undergo steep changes, such as the straightening along the tensile direction, break up GBs into small segments, and change GB structure. These changes lead to the generation of a great number of dislocations. Thus, dislocation number density increases steeply at the early stage of deformation for all the cases of lattice misfits of 4.3%, 9.2%, and 19.3%. However, after part of the original GBs has been broken, the increased rates of new dislocation slow down accordingly. Then dislocations may be absorbed into the precipitate-matrix interfaces and adjacent GBs, undergo dislocation reactions, or rearranged into new GBs. These lead to a steep decrease in dislocation dynamics in the later stage of deformation (deformation strain > 25%). The trend of dislocation density-strain curves in Figure 4 may be different from the deformation of conventional precipitation-strengthening alloys. We notice that dislocation density curves rise first and then tend to converge during the deformation of commercial precipitation-strengthening aluminum alloys [62]. The GBs between large grains with size of tens of micrometers in these alloys are very disadvantageous to absorb deformation dislocations compared with the nanocrystalline GBs in our simulations. Therefore, the dislocation density vs. strain curves in Figure 4 are consistent with the evolution of dislocation density in the deformation of nanocrystalline alloys [63].

Among the three lattice misfits, the maximum dislocation density of 4.3% lattice misfit is the largest as shown in Figure 4. The coherent precipitate-matrix interface has the lowest capacity to accommodate dislocations as mentioned above. Therefore, the maximum dislocation density decreases with the degree of lattice misfit. When lattice misfit reaches 19.3%, the incoherent phase boundary absorbs abundant dislocations, resulting in the lowest maximum value of dislocation density. For coherent and semi-coherent interfaces, dislocation density increases at the initial stage of deformation. Then, when the deformation strain increases further (deformation strain > 25%), dislocation density decreases due to the absorption of a large number of dislocations by GBs. For incoherent interface, the absorption of abundant dislocations by precipitate-matrix phase interface also contributes to the decrease of dislocation density. However, the incoherent interface has a limited ability to absorb dislocations, so dislocation density rebounds in further deformation. The time evolution of dislocation density is consistent with the dislocation dynamics in the deformation process of precipitation-strengthening alloys [62,63]. The applied stress exceeds the stress necessary for spontaneous nucleation of the dislocations, resulting in a large dislocation density value.

### 3.2. Deformation Mechanisms at Higher Strain Rate

**Figure 5:**
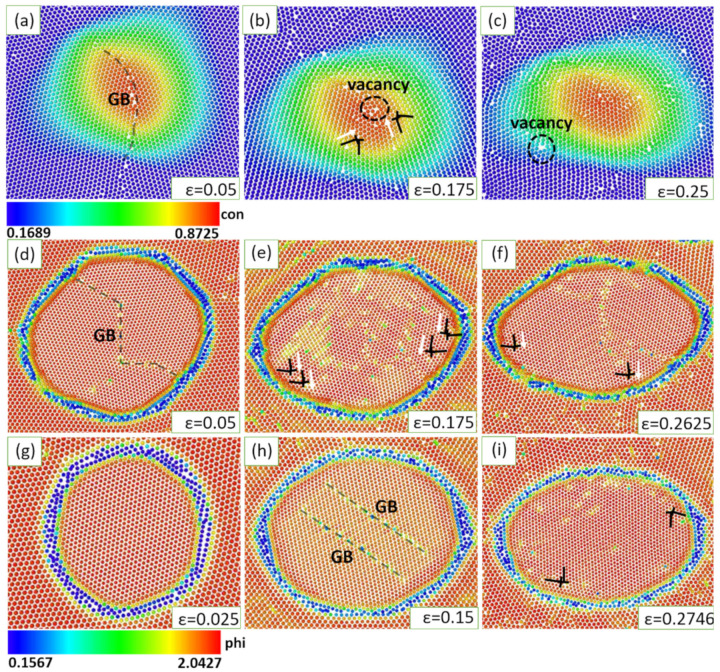
The deformation process of precipitates at a higher strain rate of $\dot{\varepsilon }$ = 10^−2^. (**a**–**c**) The evolution of the precipitate with *f* = 4.3%, the evolution of the precipitate with *f* = 19.3% containing a GB (**d**–**f**), and the evolution of the precipitate without intergranular GB with *f* = 19.3% (**g**–**i**).

**Figure 6:**
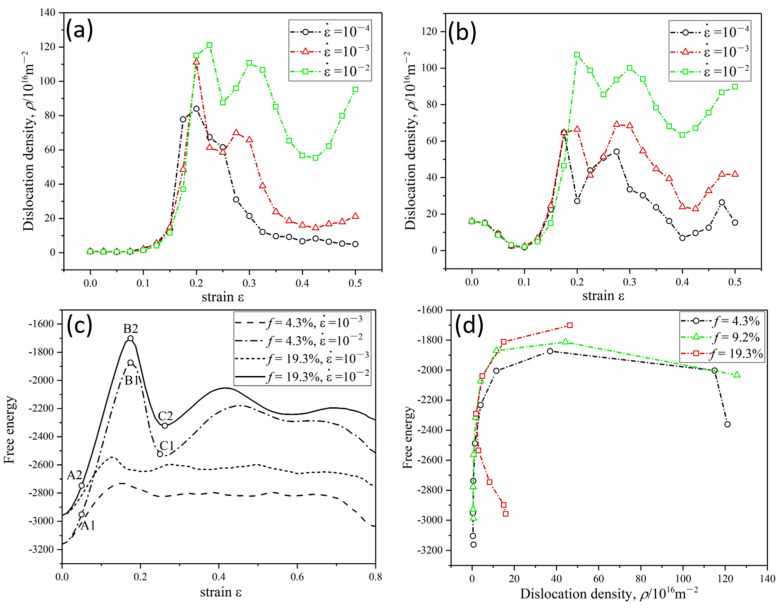
Dislocation density *ρ* and free energy evolution as a function of deformation strain *ε* for deformation processes under different degree of lattice misfit *f* and strain rates $\dot{\varepsilon }$. (**a**,**b**) the *ρ*-*ε* curves for lattice misfit *f* = 4.3% and *f* = 19.3%, respectively, (**c**) free energy-*ε* curves for deformation process, (**d**) free energy-dislocation density curves for strain rate $\dot{\varepsilon }=0.01$.

During the fast deformation process with a strain rate of 0.01, the bulk of phases, GBs, and precipitate-matrix phase interface change abruptly to release the deformation energy. In the bulk of precipitate and the grain of the matrix phase, severe lattice distortion has arisen soon at the onset of deformation. However, lattice distortion is far enough to accommodate the fast accumulated elastic energy. Then a large number of dislocations and vacancies are generated in the bulk in precipitate and grain of the matrix as shown in Figure 5. Being different from GBs slip and elongation at the strain rate of 10^−4^, the original GBs are undermined and broken into small segments, with dislocations and vacancies at a strain rate of 10^−2^. The pinned GBs are decomposed soon, leaving abundant dislocations and vacancies around the phase interface. Even for the case of a small lattice misfit of 4.3%, large lattice distortions, abundant dislocations and vacancies, and even micro-cracks could be observed in the severely deformed precipitate-matrix interface. Because of the decomposition of GBs and high dislocation density in the deformed precipitate-matrix phase interface, there is a limited space to accommodate such newly generated dislocations. Consequently, as quantified in Figure 6, the number density of dislocations of fast deformation increases to a much higher peak value than the slow deformation. The steep increases in dislocation number density contribute to the fast increase of storage energy of the system that increases with deformation rates, as shown in Figure 6c. This is in line with the classical deformation theory of alloys, which regards that the yield strength of alloys increases with deformation rates [64,65,66].

Then, the excessive dislocations would change to decrease the free energy of systems. In the slow deformations, the decrease of dislocation number could be realized by absorption into GBs and precipitate-matrix interface, or by dislocation reaction (the merge of dislocations). In the fast deformation, because of the annihilation of GBs and dislocation saturation of the precipitate-matrix interface, such as a great number of excessive dislocations have to rearrange into dislocation walls and even GBs. As shown in Figure 5f, a great number of small angle GBs are generated when the deformation strain is about 25–40%. Small grains are generated in the matrix phase. New GBs are generated, as shown in Figure 5d–f). Consequently, dislocation density decreases abruptly. This is actually dynamic recrystallization. Then, after the deformation strain increases further (deformation strain > 40%), dislocation density increases steeply due to the decomposition of newly formed GBs, and a new cycle of dynamic recrystallization starts. The research showed a dislocation density of up to 10^15^ m^−2^ after tensile deformation in aluminum alloys [66,67]. The dynamic recrystallization is not observed in the slow deformation, exemplified by the observation of dynamics of dislocations and GBs in Figure 5, and the evolution of dislocation density, free energy-strain curves, and free energy-dislocation density curves in Figure 6. The free energy of the system is calculated as the current free energy minus the free energy of the reference state, with negative values indicating a decrease in energy relative to the reference state [44,45]. The energy of the regularly arranged crystal is the lowest, and the propagation of new dislocations produces additional free energy. As shown in Figure 6d, dislocation density is proportional to free energy in the initial deformation stage.

### 3.3. Deformation Mechanisms of Precipitate-Matrix Phase Interface

Finally, we observed that the three kinds of precipitate-matrix phase interfaces, including coherent, semi-coherent, and incoherent interfaces, show very different deformation behaviors. As shown in Figure 2, lattice distortion could be released with the help of the evolution of GBs during the slow deformation process. As the deformation rate increases, in order to releases elastic energy more efficiently, severe lattice distortion occurs, and then dislocations are generated. Generally, the coherent phase interface shows no essential difference with the bulk of precipitate and matrix phases during slow and fast deformation processes.

**Figure 7:**
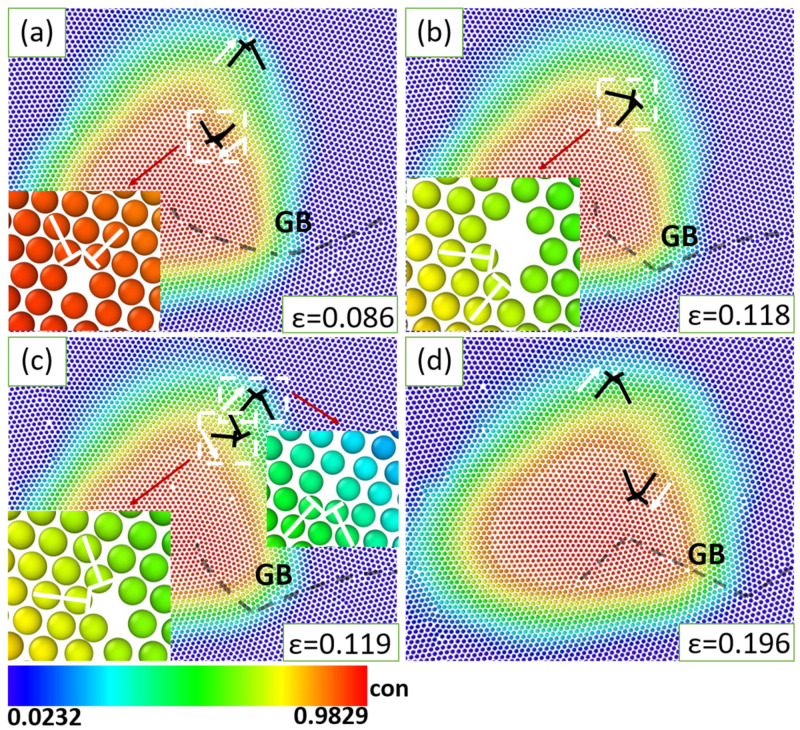
Emitting and separation of dislocation pairs at the semi-coherent interface of different deformation strains during deformation with a strain rate $\dot{\varepsilon }$ = 10^−4^, (**a**) *ε =* 0.086, (**b**) *ε* = 0.118, (**c**) *ε* = 0.119, (**d**) *ε* = 0.196. The white arrows mark the moving directions of dislocation, the red arrows are signs.

The semi-coherent precipitate-matrix interface behaves different from the coherent interface. As shown in Figure 7, even in the slow deformation process, we observed that pairs of dislocations are continuously generated at the phase interface, and move in opposite directions, with one moving away into the matrix phase, and the other passing through the precipitate and being absorbed by the GB pinned by the precipitate. This is because larger lattice misfit between the precipitate and matrix phases could produce more misfit strain during deformation. Driven by the decrease of misfit strain, the pair of dislocations move like the dynamics of misfit dislocations in the phase interface during epitaxial growth [68]. Nevertheless, during fast deformation, the large lattice distortion-dislocation generation regimen dominates over the generation of dislocation pairs. The incoherent phase interface does not produce excess dislocations to release elastic energy because of its loose and disordered structures; instead, they act as the sink of moving dislocations as we introduced above.

## 4. Conclusions

In this study, the PFC simulations were used to investigate in detail the precipitate-GB interactions and dislocation-GB/precipitate interactions of deforming precipitation-strengthening alloys. The main conclusions are as follows:

In slow deformation with a strain rate of 10^−4^, the pinning effect of precipitate can actually hinder the motion of deforming GBs, and becomes increasingly strong with the increase of lattice misfit. In the faster deformation with a strain rate of 10^−2^, the original GBs are undermined and broken into small segments, dislocations, and vacancies. Therefore, the dislocation density of fast deformation is higher.

Lattice misfit between the precipitate and matrix phase could also strongly affect the deformation behaviors of alloys in conventional slow deformations. The cut regimen prevails under the interaction between coherent precipitate and dislocations; the dislocations move toward the GBs and are finally absorbed. In the case of lattice misfit of 19.3%, the dislocations tend to move to the incoherent phase boundary and be absorbed. Collaborative deformation is observed in coherent and semi-coherent interfaces in the slow deformation. Interestingly, semi-coherent interface has to emit pairs of dislocations continuously to coordinate the deformation of the abutting precipitate and matrix grain. Incoherent precipitate deforms independently from the matrix grain.

Taken as a whole, the results contribute to important insights into the understanding on the fundamental questions that how the microstructure of precipitation strengthening alloys, including nano-precipitates, matrix grains, and precipitate-matrix phase interface deform collaboratively or independently under different lattice misfits and deformation rates.

## Figures and Tables

**Table 1:** Parameters of PFC simulation.

Parameters	Symbols	Values/Expressions
Reference density	$\rho^{0}$	0.01
Reference composition	c_0_	0.5
Polynomial fitting parameters	η, χ	η= 1.4, χ= 1
Entropy of mixing coefficient	ω	0.005
Parameters for correlation function	$\sigma_{M_{j}},\alpha_{i}$	$\sigma_{M_{j}}=$0.8, $\alpha_{i}=2.0$
Gradient energy coefficient	α	1

## Data Availability

The data that support the findings of this study are available from the authors upon reasonable request.

## Supplementary Materials

The following supporting information can be downloaded at: https://www.mdpi.com/article/10.3390/ma16051841/s1, Video S1: The evolution of precipitation process with lattice misfit of 4.3%; Video S2: The evolution of precipitation process with lattice misfit of 9.2%; Video S3: The evolution of precipitation process with lattice misfit of 19.3%. Videos S4–S6: The interactions between GB and precipitate during slow deformation with strain rate $\dot{\varepsilon }$ = 10^−4^ with lattice misfit of *f* = 4.3%, *f* = 9.2%, *f* = 19.3% respectively.

## References

[1] Wei X., Cao X., Luan J., Jiao Z., Liu C., Zhang Z. (2022). Synergy of strengthening and toughening of a Cu-rich precipitate-strengthened steel. Mater. Sci. Eng. A.

[2] Wang Q., Li Z., Pang S., Li X., Dong C., Liaw P.K. (2018). Coherent Precipitation and Strengthening in Compositionally Complex Alloys: A Review. Entropy.

[3] Xiong Z., Timokhina I., Pereloma E. (2020). Clustering, nano-scale precipitation and strengthening of steels. Prog. Mater. Sci..

[4] Stornelli G., Gaggia D., Rallini M., Di Schino A. (2021). Heat treatment effect on maraging steel manufactured by laser powder bed fusion technology: Microstructure and mechanical properties. Acta Met. Slovaca.

[5] Xu S., Zhao Y., Chen D., Sun L., Chen L., Tong X., Liu C., Zhang Z. (2018). Nanoscale precipitation and its influence on strengthening mechanisms in an ultra-high strength low-carbon steel. Int. J. Plast..

[6] Chambrin N., Dalverny O., Cloue J.-M., Brucelle O., Alexis J. (2022). In Situ Ageing with the Platform Preheating of AlSi10Mg Alloy Manufactured by Laser Powder-Bed Fusion Process. Metals.

[7] Marlaud T., Deschamps A., Bley F., Lefebvre W., Baroux B. (2010). Influence of alloy composition and heat treatment on precipitate composition in Al-Zn-Mg-Cu alloys. Acta Mater..

[8] Lee S.-H., Jung J.-G., Baik S.-I., Seidman D., Kim M.-S., Lee Y.-K., Euh K. (2021). Precipitation strengthening in naturally aged Al-Zn-Mg-Cu alloy. Mater. Sci. Eng. a-Struct. Mater. Prop. Microstruct. Process..

[9] Rios P., Fonseca G. (2004). Grain boundary pinning by Al6Mn precipitates in an Al–1wt%Mn alloy. Scr. Mater..

[10] Wang N., Ji Y., Wang Y., Wen Y., Chen L.-Q. (2017). Two modes of grain boundary pinning by coherent precipitates. Acta Mater..

[11] Ahmadi M., Povoden-Karadeniz E., Öksüz K., Falahati A., Kozeschnik E. (2014). A model for precipitation strengthening in multi-particle systems. Comput. Mater. Sci..

[12] Randle V., Ralph B. (1986). Interactions of grain boundaries with coherent precipitates during grain growth. Acta Met..

[13] Bai S., Di H., Liu Z. (2016). Dislocation interaction with Omega phase in crept Al-Cu-Mg-Ag alloys. Mater. Sci. Eng. A-Struct. Mater. Prop. Microstruct. Process..

[14] Hirouchi T., Takaki T., Tomita Y. (2010). Effects of temperature and grain size on phase-field-crystal deformation simulation. Int. J. Mech. Sci..

[15] Williams K.R., Fisher S.B. (1975). The interaction of dislocation loop and γ’ precipitate in nickel based alloys. Radiat. Eff..

[16] Bahl S., Xiong L., Allard L., Michi R., Poplawsky J., Chuang A., Singh D., Watkins T., Shin D., Haynes J. (2021). Aging behavior and strengthening mechanisms of coarsening resistant metastable theta’ precipitates in an Al-Cu alloy. Mater. Des..

[17] Gutierrez-Urrutia I., Zaefferer S., Raabe D. (2013). Coupling of Electron Channeling with EBSD: Toward the Quantitative Characterization of Deformation Structures in the SEM. Jom.

[18] Kobayashi M., Miura H. (2020). 3D/4D characterization of strain distribution evolving within the microstructure during plastic deformation. Curr. Opin. Solid State Mater. Sci..

[19] Yang J., Liu C., Ma P., Chen L., Zhan L., Yan N. (2022). Superposed hardening from precipitates and dislocations enhances strength-ductility balance in Al-Cu alloy. Int. J. Plast..

[20] Chrominski W., Lewandowska M. (2020). Influence of dislocation structures on precipitation phenomena in rolled Al-Mg-Si alloy. Mater. Sci. Eng. a-Struct. Mater. Prop. Microstruct. Process..

[21] Liao Y., Ye C., Gao H., Kim B.-J., Suslov S., Stach E.A., Cheng G.J. (2011). Dislocation pinning effects induced by nano-precipitates during warm laser shock peening: Dislocation dynamic simulation and experiments. J. Appl. Phys..

[22] Shaha S., Czerwinski F., Kasprzak W., Friedman J., Chen D., Shalchi-Amirkhiz B. (2017). Interaction between nano-precipitates and dislocations during high temperature deformation of Al-Si alloys. J. Alloy. Compd..

[23] Baruah M., Borah A. (2020). Processing and precipitation strengthening of 6xxx series aluminium alloys: A review. Int. J. Mater. Sci..

[24] Tan F., Fang Q., Li J., Wu H. (2019). Interaction of precipitate with shear–coupled grain boundary migration. Acta Mech..

[25] Marenych O., Kostryzhev A. (2020). Strengthening Mechanisms in Nickel-Copper Alloys: A Review. Metals.

[26] Cepeda-Jiménez C., Castillo-Rodríguez M., Pérez-Prado M. (2018). Origin of the low precipitation hardening in magnesium alloys. Acta Mater..

[27] Mukherji D., Gilles R., Barbier B., Del Genovese D., Hasse B., Strunz P., Wroblewski T., Fuess H., Rösler J. (2003). Lattice misfit measurement in Inconel 706 containing coherent γ′ and γ″ precipitates. Scr. Mater..

[28] Thompson A., Brooks J. (1982). The mechanism of precipitation strengthening in an iron-base superalloy. Acta Met..

[29] Peng J., Li Z., Fu L., Ji X., Pang Z., Shan A. (2019). Carbide precipitation strengthening in fine-grained carbon-doped FeCoCrNiMn high entropy alloy. J. Alloy. Compd..

[30] Lai Y., Fan W., Yin M., Wu C., Chen J. (2019). Structures and formation mechanisms of dislocation-induced precipitates in relation to the age-hardening responses of Al-Mg-Si alloys. J. Mater. Sci. Technol..

[31] Biswas A., Siegel D.J., Wolverton C., Seidman D.N. (2011). Precipitates in Al–Cu alloys revisited: Atom-probe tomographic experiments and first-principles calculations of compositional evolution and interfacial segregation. Acta Mater..

[32] Maciejewski K., Jouiad M., Ghonem H. (2013). Dislocation/precipitate interactions in IN100 at 650 °C. Mater. Sci. Eng. A.

[33] Liu H., Gao Y., Qi L., Wang Y., Nie J.-F. (2015). Phase-Field Simulation of Orowan Strengthening by Coherent Precipitate Plates in an Aluminum Alloy. Met. Mater. Trans. A.

[34] Li J., Chen H., Fang Q., Jiang C., Liu Y., Liaw P.K. (2020). Unraveling the dislocation–precipitate interactions in high-entropy alloys. Int. J. Plast..

[35] Liao M., Li B., Horstemeyer M.F. (2014). Interaction Between Basal Slip and a Mg17Al12 Precipitate in Magnesium. Met. Mater. Trans. A.

[36] Huo J.-R., Yang H.-Y., Wang J., He C.-Z. (2021). Computational simulation of al-based alloy surface structure dislocation: The first-principles calculation and atomic pair-potential lattice dynamics calculation. Mod. Phys. Lett. B.

[37] Wu K.-A., Voorhees P.W. (2012). Phase field crystal simulations of nanocrystalline grain growth in two dimensions. Acta Mater..

[38] Yamanaka A., McReynolds K., Voorhees P.W. (2017). Phase field crystal simulation of grain boundary motion, grain rotation and dislocation reactions in a BCC bicrystal. Acta Mater..

[39] Zhao Y., Liu K., Zhang H., Tian X., Jiang Q., Murugadoss V., Hou H. (2022). Dislocation motion in plastic deformation of nano polycrystalline metal materials: A phase field crystal method study. Adv. Compos. Hybrid Mater..

[40] Gao Y., Huang L., Deng Q., Zhou W., Luo Z., Lin K. (2016). Phase field crystal simulation of dislocation configuration evolution in dynamic recovery in two dimensions. Acta Mater..

[41] Fallah V., Korinek A., Ofori-Opoku N., Raeisinia B., Gallerneault M., Provatas N., Esmaeili S. (2015). Atomic-scale pathway of early-stage precipitation in Al-Mg-Si alloys. Acta Mater..

[42] Fallah V., Ofori-Opoku N., Stolle J., Provatas N., Esmaeili S. (2013). Simulation of early-stage clustering in ternary metal alloys using the phase-field crystal method. Acta Mater..

[43] Elder K.R., Provatas N., Berry J., Stefanovic P., Grant M. (2007). Phase-field crystal modeling and classical density functional theory of freezing. Phys. Rev. B.

[44] Greenwood M., Ofori-Opoku N., Rottler J., Provatas N. (2011). Modeling structural transformations in binary alloys with phase field crystals. Phys. Rev. B.

[45] Greenwood M., Rottler J., Provatas N. (2011). Phase-field-crystal methodology for modeling of structural transformations. Phys. Rev. E.

[46] Huang Z.-F., Elder K., Provatas N. (2010). Phase-field-crystal dynamics for binary systems: Derivation from dynamical density functional theory, amplitude equation formalism, and applications to alloy heterostructures. Phys. Rev. E.

[47] Ofori-Opoku N., Fallah V., Greenwood M., Esmaeili S., Provatas N. (2013). Multicomponent phase-field crystal model for structural transformations in metal alloys. Phys. Rev. B.

[48] Granasy L., Tegze G., Toth G., Pusztai T. (2011). Phase-field crystal modelling of crystal nucleation, heteroepitaxy and patterning. Philos. Mag..

[49] Fallah V., Korinek A., Ofori-Opoku N., Provatas N., Esmaeili S. (2013). Atomistic investigation of clustering phenomenon in the Al–Cu system: Three-dimensional phase-field crystal simulation and HRTEM/HRSTEM characterization. Acta Mater..

[50] Stefanovic P., Haataja M., Provatas N. (2009). Phase field crystal study of deformation and plasticity in nanocrystalline materials. Phys. Rev. E.

[51] Shuai X., Wang Z.J., Mao H., Tang S., Kong Y., Du Y. (2021). Atomic-scale study of compositional and structural evolution of early-stage grain boundary precipitation in Al–Cu alloys through phase-field crystal simulation. J. Mater. Sci..

[52] Shuai X., Mao H., Tang S., Kong Y., Du Y. (2022). Growth modes of grain boundary precipitate in aluminum alloys under different lattice misfits. J. Mater. Sci..

[53] Dehghan-Manshadi A., Dippenaar R. (2010). The Behavior of Precipitates during Hot-Deformation of Low-Manganese, Titanium-Added Pipeline Steels. Metall. Mater. Trans. a-Phys. Metall. Mater. Sci..

[54] Gong X., Luo S., Li S., Wu C. (2021). Dislocation-Induced Precipitation and Its Strengthening of Al-Cu-Li-Mg Alloys with High Mg. Acta Metall. Sin. -Engl. Lett..

[55] Chen L., Shen J. (1998). Applications of semi-implicit Fourier-spectral method to phase field equations. Comput. Phys. Commun..

[56] Hirouchi T., Takaki T., Tomita Y. (2009). Development of numerical scheme for phase field crystal deformation simulation. Comput. Mater. Sci..

[57] Sakaguchi N., Shibayama T., Kinoshita H., Takahashi H. (2002). Atomistic dynamical observation of grain boundary structural changes under electron irradiation. J. Nucl. Mater..

[58] Bernstein N. (2008). The influence of geometry on grain boundary motion and rotation. Acta Mater..

[59] Tayon W.A., Nygren K.E., Crooks R.E., Pagan D.C. (2019). In-situ study of planar slip in a commercial aluminum-lithium alloy using high energy X-ray diffraction microscopy. Acta Mater..

[60] Zhu A., Chen J., Starke E. (2000). Precipitation strengthening of stress-aged Al-xCu alloys. Acta Mater..

[61] Wang Z., Zhang J., Lu J. (2022). Atomic-scale study of dislocation-grain boundary interactions in Cu bicrystal by Berkovich nanoindentation. Mater. Sci. Eng. A.

[62] Bratov V., Borodin E. (2015). Comparison of dislocation density based approaches for prediction of defect structure evolution in aluminium and copper processed by ECAP. Mater. Sci. Eng. A.

[63] Ni S., Wang Y., Liao X., Alhajeri S., Li H., Zhao Y., Lavernia E., Ringer S., Langdon T., Zhu Y. (2011). Grain growth and dislocation density evolution in a nanocrystalline Ni–Fe alloy induced by high-pressure torsion. Scr. Mater..

[64] Tandon R., Mehta K., Manna R., Mandal R. (2022). Effect of tensile straining on the precipitation and dislocation behavior of AA7075T7352 aluminum alloy. J. Alloy. Compd..

[65] Lee W.-S., Chen T.-H. (2006). Rate-dependent deformation and dislocation substructure of Al–Sc alloy. Scr. Mater..

[66] Thirathipviwat P., Nozawa S., Furusawa M., Onuki Y., Hasegawa M., Matsumoto K., Sato S. (2022). In-situ neutron diffraction study on a dislocation density in a correlation with strain hardening in Al–Mg alloys. Mater. Sci. Eng. A.

[67] Zuiko I., Kaibyshev R. (2017). Deformation structures and strengthening mechanisms in an Al-Cu alloy subjected to extensive cold rolling. Mater. Sci. Eng. A.

[68] Jia B.W., Tan K.H., Loke W.K., Wicaksono S., Yoon S.F. (2017). Effects of surface reconstruction on the epitaxial growth of III-Sb on GaAs using interfacial misfit array. Appl. Surf. Sci..

